# Excess All-Cause Mortality During the COVID-19 Outbreak in Japan

**DOI:** 10.2188/jea.JE20200492

**Published:** 2021-01-05

**Authors:** Takashi Yorifuji, Naomi Matsumoto, Soshi Takao

**Affiliations:** Department of Epidemiology, Graduate School of Medicine, Dentistry and Pharmaceutical Sciences, Okayama University, Okayama, Japan

Dear Sir

The outbreak of coronavirus disease 2019 (COVID-19) began in Wuhan, China, in December 2019, and is now a global pandemic.^1^ COVID-19 has placed a huge burden on population health. One of the indicators used to assess the impact is excess mortality. Excess mortality includes mortality of people diagnosed with COVID-19, those infected with COVID-19 but undiagnosed, and those who were not infected with COVID-19 but died from social changes caused by the pandemic. Marked excess mortality has already been reported in the US and Europe.^2^^–^^4^ Japan is also experiencing a COVID-19 outbreak, but the impact seems to be minimal (1,601 deaths in a population of 126 million as of 6th October 2020).^5^ Assessment of excess mortality may enable more detailed evaluation of the impact of COVID-19. Here, we evaluated the excess mortality during the COVID-19 epidemic in Japan.

The study period was from January 2020, when the Government strengthened public health regulations such as hand washing against infections including COVID-19, to July 2020, when the latest mortality data were available. We obtained the number of patients with COVID-19 per day in Japan, the cumulative number of patients with COVID-19 by prefecture up to 31^st^ July 2020, and mortality data (2018–2020) from the Prompt Vital Statistics Report from the Ministry of Health, Labour and Welfare in Japan.

The monthly all-cause excess mortality (number) from January to July 2020 was estimated by subtracting the expected monthly mortality (mean monthly mortality in 2018 and 2019) from the observed mortality of the corresponding month in 2020. We then plotted the monthly excess mortality with the number of patients with COVID-19 per day in Japan. We also evaluated the prefectural-level association between the cumulative incidence of patients with COVID-19 up to July 2020 and the excess mortality rate from January to July 2020. We used the population estimation in October 2019 from the Statistic Bureau of Japan to calculate the cumulative incidence and excess mortality rate. We calculated the correlation coefficient using Stata 16 (StataCorp, College Station, TX, USA). Ethical approval was not obtained because we used aggregate data from publicly available sources.

**Figure 1.**
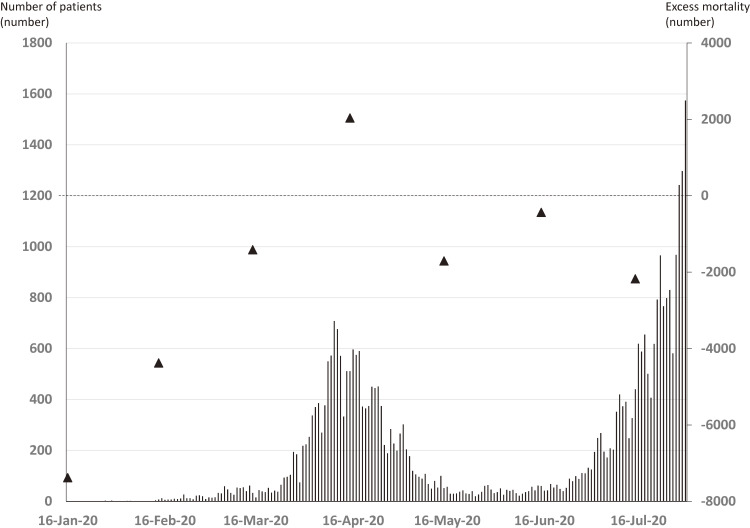
Daily number of patients with COVID-19 and monthly excess mortality in Japan (▲).

**Figure 2.**
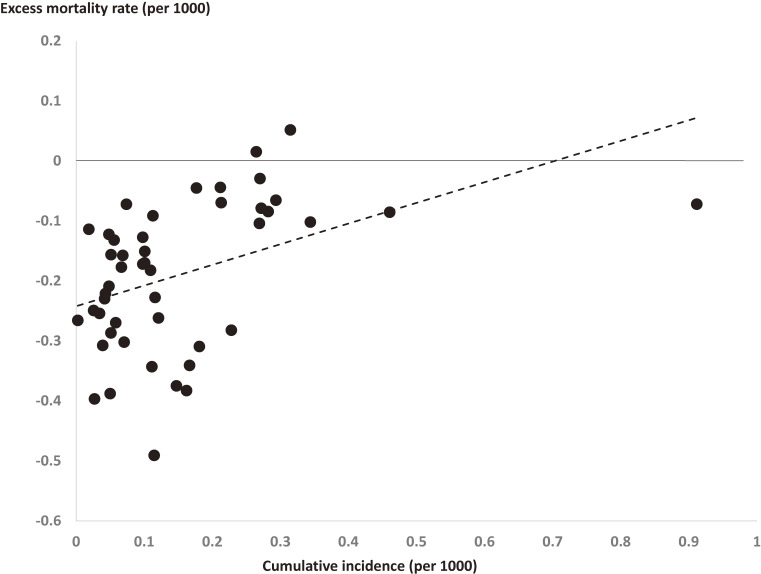
Cumulative number of patients with COVID-19 in Japan until July 2020, and excess mortality from January to July 2020 in each prefecture.

There were 132,622, 117,010, 119,161, 113,362, 108,380, 100,423, and 104,849 total deaths per month in Japan from January to July 2020. The first patient in Japan with COVID-19 was identified in January, and the number of patients peaked in mid-April and in July (Figure 1). The excess mortality (number) was positive only in April (about 2,000 excess deaths), but was negative for the other months during the study period. Prefectures with a higher COVID-19 incidence tended to have an increased excess mortality rate (*r* = 0.43); however, there was a negative excess mortality rate in all except two prefectures (Figure 2).

**Figure 3.**
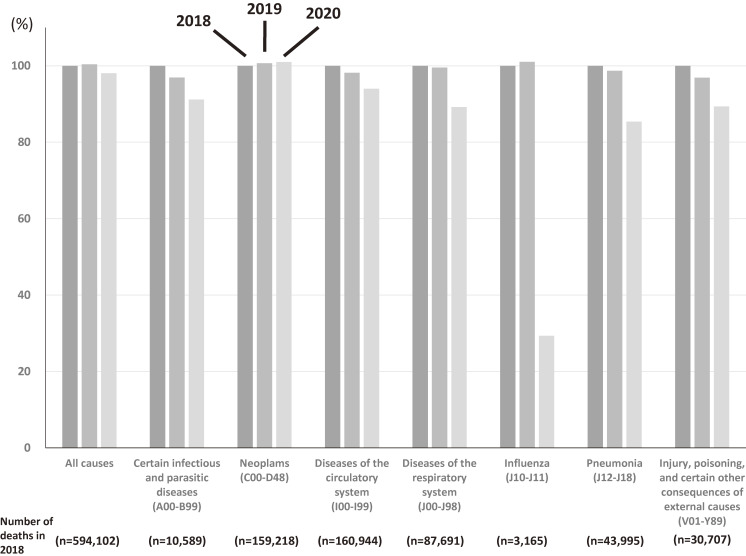
Percent change relative to cumulative number of mortality from January to May in 2018 separated by selected causes of death during the period from 2018 to 2020 in Japan. ICD-10 (International Classification of Diseases 10th Revision) codes are shown in the parentheses. Numbers of deaths from January to May in 2018 are also shown in the bottom.

These results demonstrate that excess mortality in Japan occurred only in April 2020 (at the first peak of the COVID-19 outbreak). In contrast, the numbers were negative in the other assessed months. Overall, therefore, net mortality decreased during the study period compared with the previous 2 years (2018 and 2019). Moreover, although there was a positive prefecture-level relationship between cumulative COVID-19 incidence and excess mortality rate, most prefectures had a negative excess mortality rate. We obtained the cause-specific cumulative number of mortalities from January to May in Japan (2018–2020), when the latest cause-specific mortality data were available, from the Monthly Vital Statistics Report from the Ministry of Health, Labour and Welfare in Japan. We then show the percent change relative to the number of deaths in 2018 in each year separated by selected causes of death (Figure 3). This figure shows that the decreased monthly and net mortality are partly explained by decreased mortality related to infection, including influenza^6^ or pneumonia, owing to the reduced transmission risk, accidents possibly due to the lower social activity,^7^ or circulatory diseases. Although a limitation of our study is that we did not consider excess mortality during the second wave of COVID-19 infections in July/August 2020, the results suggest that public health regulations aimed at preventing COVID-19 may incidentally reduce mortality related to influenza and other infections or other causes and consequently contribute to reduced net mortality.
